# Value of Including the Children’s Experience for Improving Their Rights During Hospitalization: Protocol for the VoiCEs Project

**DOI:** 10.2196/42804

**Published:** 2023-04-03

**Authors:** Sabina De Rosis, Manila Bonciani, Veronica Spataro, Ilaria Corazza, Elisa Conti, Barbara Sibbles, Jan A Hazelzet, Pekka Lahdenne, Katariina Gehrmann, Francesca Menegazzo, Michela Sica, Vita Šteina, Guna Esenberga, Elise M Chapin, Stefania Solare, Milena Vainieri

**Affiliations:** 1 Management and Healthcare Laboratory, Institute of Management, Sant’Anna School of Advanced Studies Pisa Italy; 2 Meyer Children’s Hospital IRCCS Florence Italy; 3 Department of Public Health, Erasmus University Medical Center Rotterdam Netherlands; 4 Helsinki University Hospital, Children’s Hospital Helsinki Finland; 5 Children's Clinical University Hospital Rīga Latvia; 6 Italian National Committee for UNICEF Rome Italy

**Keywords:** patient experience, patient-reported experience, PREM, pediatric patients, children, adolescents, coproduction, coassessment, health care services, hospitalization

## Abstract

**Background:**

Users’ feedback is a key asset for organizations that want to improve their services. Studying how organizations are enabling their users to participate in evaluation activities is particularly important, especially when there are vulnerable or disadvantaged people, and the services to be evaluated can be life-changing. This is the case in the coassessment by pediatric patients experiencing hospital stay. The international literature reports a few attempts and several challenges in systematically collecting and using the pediatric patient experience with respect to hospitalization, to undertake quality improvement actions.

**Objective:**

This paper describes the research protocol of a European project intended to develop and implement a systematic pediatric patient-reported experience measures (PREMs) observatory that will be shared by 4 European children’s hospitals in Finland, Italy, Latvia, and the Netherlands.

**Methods:**

The VoiCEs (Value of including the Children’s Experience for improving their rightS during hospitalization) project uses a participatory action research approach, based on a mixture of qualitative and quantitative methods. It consists of 6 different phases, including a literature review, an analysis of the previous experiences of pediatric PREMs reported by project partners, a Delphi process, a cycle of focus groups or in-depth interviews with children and their caregivers, a series of workshops with interactive working groups, and a cross-sectional observational survey. The project guarantees the direct participation of children and adolescents in the development and implementation phases of the project.

**Results:**

The expected results are (1) a deeper knowledge of published methodologies and tools on collecting and reporting pediatric patients’ voice; (2) lessons learnt from the analysis of previous experiences of pediatric PREMs; a consensus reached through a participatory process (3) among experts, (4) pediatric patients and caregivers about a standard set of measures for the evaluation of hospitalization by patients; (5) the implementation of a European observatory on pediatric PREMs; and (6) the collection and comparative reporting of the pediatric patients’ voice. In addition, the project is aimed at studying and proposing innovative methodologies and tools for capturing the pediatric patients’ feedback directly, avoiding the intermediation of parents/guardians.

**Conclusions:**

Over the last decade, the collection and use of PREMs have gained importance as a research field. Children and adolescents’ perspectives have also been increasingly taken into consideration. However, to date, there are limited experiences regarding the continuous and systematic collection and use of pediatric PREMs data for implementing timely improvement actions. In this perspective, the VoiCEs project provides room for innovation, by contributing to the creation of an international, continuous, and systematic pediatric PREMs observatory that can be joined by other children’s hospitals or hospitals with pediatric patients, and foresees the return of usable and actionable data in benchmarking.

**International Registered Report Identifier (IRRID):**

DERR1-10.2196/42804

## Introduction

### Context

Children are one of the most fragile segments of the population and they might be disadvantaged in expressing their opinions, experiences, or preferences. As stated by the Manual for Human Rights Education with Young People, “the child has the right to express freely views on all matters affecting him/her, and the child’s views should be given due weight.” Great efforts have been made in Europe to strengthen “the right of the child to be heard” [1]. Recently, the European Union (EU) and the UNICEF (United Nations International Children's Emergency Fund/United Nations Children’s Fund) promoted #TheRealChallenge campaign to raise attention on this right and make children express their views [2]. The European Youth and Information Counselling Agency (ERYICA) aims to uphold the right of young people to be empowered, make informed choices, and be active in society [3]. The project GIOCONDA financed under the LIFE+ program involves young people as protagonists of an action of participative democracy, with special attention to environment and health policies [4]. The Council of Europe “Guidelines on Child-Friendly Health Care” has promoted the design of health care services centered on children’s rights and needs, considering their opinion and easing their participation. The World Health Organization (WHO) has also promoted a tool to assess the quality of hospital care provided to children in a systematic and participatory way, as well as children’s rights in a hospital [5,6].

Literature and practice are demonstrating the centrality of listening to children and young people’s voices on health care, both to increase the awareness of how this vulnerable population experiences care and to improve health care policy and practice [7]. Coproduction is among the most promising forms of participation with young people [8].

### Current Knowledge and Previous Experiences

Evaluation of health care services is a coassessment process including comonitoring and coevaluating activities of lay people [9]. The most used definition of coproduction in the public sector defines coassessment as being focused “on monitoring and evaluating public services where state and lay actors work together to assess service quality, problems, and/or areas for improvement” [10, p. 772]. Consequently, coassessment usually happens retrospectively, when the service has been already experienced, but with the possibility of prospectively affecting the future design or delivery of the services [10]. The use of coassessment results can inform co-design and coinnovation processes aimed at improving or innovating services [11].

The participation of lay actors in coassessment processes remains rare in several sectors [12-14]. Strokosch and Osborne [12] described the negative experiences of assessment processes involving people with disability in the United Kingdom, by either public social and health care organizations or for-profit organizations. Loeffler and Timm-Arnold [13] reported a low or bad level of public involvement in coassessment initiatives concerning public safety and social services not only for young people, but also for elderly people, both using digital channels and traditional methods. Indeed, the level of participation depends also on the successful design and implementation of these coassessment initiatives. Among the most important antecedents of public participation in the evaluation of services, the so-called opportunities are those more closely linked to the service organization’s activities. Organizations create opportunity of public participation by facilitating the citizens’ engagement in the processes; by increasing the people’s ability to effectively coproduce; by reducing the difficulties of participation [15,16]; or by setting motivational incentives, nudges, and other levers of participation [17,18]. Pestoff [19] referred to the “ease of involvement,” which encompasses the cost of the involvement, the professional support including capacity building and direct mobilization of coproducers, and the definition of motivational incentives and nudges. Working on people’s ability encompasses investigating these factors, which include human capital and socioeconomic conditions [20], individual characteristics (ie, age, sex, educational level), and being a user of the service to be evaluated as a factor affecting the internal efficacy of people, namely, how much they feel able to participate in certain activities, as well as their engagement [21].

From this perspective, studying how health care organizations can facilitate public participation in coassessment of services is extremely relevant. This is particularly important when the application of such research activities concerns the participation of vulnerable or disadvantaged people, and the services to be evaluated can be life changing. This is the case of the coassessment by pediatric patients experiencing hospitalization. As hospitalization represents a very delicate moment, it is especially important to focus on health-related vulnerable children and adolescents. This research project focuses on raising children’s and adolescents’ voices on their experience with hospital care, by studying and implementing opportunities for them to coassess and, consequently, contribute to the improvement of hospitalization services.

There are several experimental or cross-sectional studies aimed at investigating children’s perceptions during hospitalization [22-25]. Using the Hospital Consumer Assessment of Healthcare Providers and Systems Survey (HCAHPS) [26] and the Picker Institute [27] questionnaires, health care organizations or systems periodically collect measures of experience with services, as perceived by samples of patients. At the EU level, only sporadic international attempts were found in the literature on children’s experience of hospitalization, which aimed at detecting their voices on a census-like, continuous, and systematic basis [28,29]. Nevertheless, previous experiences were mainly time-constrained and did not include systematic data collection. Moreover, they were mainly sample based, not involving potentially all children, without a systematized cross-border perspective and impact.

Although children are one of the most fragile segments of the population and the hospitalization experience can per se make them more vulnerable, usually hospitalized children do not have a chance to express an opinion about the care they received. Conversely, practitioners should make decisions considering children’s preferences and opinions. To this end, it is necessary to develop a tool able to give pediatric patients the opportunity to make their voices heard about such delicate moments. Developing a common and scalable observatory that continuously collects data on children’s and parent’s experiences with hospital care across the EU is an ambitious goal that allows the children’s right of expression to be guaranteed. To the best of our knowledge, there is no European standard on patient-reported experience measures (PREMs) for children, nor an example of pediatric PREMs observatory that can be joined by European and non-European countries in the future, for collecting and benchmarking the pediatric patient voice.

This protocol presents the VoiCEs (Value of including the Children’s Experience for improving their rightS during hospitalization) project, which represents a fundamental step toward the improvement of children’s rights of coassessing and coinnovating health care services, as well as of practitioners’ attention on their young patients’ rights. This paper presents the (1) settings, (2) aims, (3) methods and analysis, and (4) ethical aspects of the VoiCEs project.

### Setting

The project will be conducted by a public university in Italy, which is the principal investigator (PI) and works with a dedicated research team.

A total of 4 children’s clinical university hospitals from different European countries will be directly involved as project partners:

- Meyer Children’s University Hospital, Florence, Italy (AOU Meyer)
- Children’s Clinical University Hospital, Riga, Latvia (CCUH)
- HUS New Children’s Hospital, Helsinki, Finland (HUS)
- Erasmus University Medical Center, Sophia Children’s Hospital, Rotterdam, The Netherlands (EMC)

The 4 countries cover the European south-north-east-west areas, which will ease the development of a scalable model at the European level. The 4 hospitals involved have already worked on the collection of hospitalized children’s voices. In the Finnish project LAPSUS, digital methods have been piloted to capture experiences from children and teenagers [30]. The children’s hospitals in Florence (Italy) and Riga (Latvia), and several university children’s hospitals in the Netherlands as well, adopted PREMs for children’s hospital care [28,29,31,32], reporting results on web platforms to support children-driven improvements [28,29,33,34]. These valuable experiences achieved good results in terms of child participation, and they will be the starting point to design a new European tool.

To this end, the participation within the consortium of the UNICEF, with its international and local experience on the promotion of children’s rights, will ensure the focus on the priority, synergies with other European projects concerning children’s rights, and a wider impact of the project.

This positive impact at the European level will be also enhanced by the support to the project from other organizations, such as:

- The European Children’s Hospitals Organisation (ECHO), which supports cross-border collaborative actions and learning processes for promoting children’s rights in European hospitals and improving health and care for pediatric patients;
- The Italian Association of Children’s Hospitals (AOPI), which will help further promote the results among the children’s hospitals in Italy;
- The Picker Institute, whose competence and interest on patient experience will contribute to increase the strength of the scientific approach adopted in the project and support a wider dissemination of its results.

### Aims

The aim of the VoiCEs project is to design and implement a system ensuring that hospitalized children can continuously be involved and heard. This would help identify which aspects of care in pediatric hospitals should be improved from their point of view. The long-term objective is to make inpatient care more child-friendly in children’s hospitals.

The specific objectives to be reached within the duration of the project are as follows:

- To develop a common list of measures and questions to collect the experience of pediatric patients and their parents, by directly involving experts among researchers, hospital staff and stakeholders, and children from the 4 European countries of the participating children’s hospitals;
- To implement and to provide hospitalized children a mechanism to express their feelings and perceptions about their hospitalization experience in a continuous way;
- To enhance the attention of children’s hospital professionals on the relevance of hearing and taking into consideration the voices of their pediatric patients in evaluating and improving services;
- To provide hospital professionals with guidelines for defining information material and organizing training programs, with the aim of promoting children’s rights to make their voices heard on the experience of hospitalization; and using children’s feedback for supporting improvement actions.

The project will contribute to enhancing children’s participation in the evaluation and improvement of health care services during hospitalization. This will be done with the implementation of a common PREMs tool and benchmarking system in the 4 children’s hospitals and with the planning of quality improvement activities. At the local level, one of the expected results is the increased awareness among hospital staff, as well as external stakeholders, of the relevance of listening to the voices of children as patients. This makes it possible to ensure high-quality health care services and a better experience during hospitalization. Another important outcome that can be expanded to a national level will be the benefits of child-friendly care developed using the results of the PREMs observatory. The middle-term impact will be the improvement of children’s rights in the setting of the 4 children’s hospitals involved in the project. At the European level, the project will make a common tool available to collect the children’s experience of hospitalization, such as the validated PREMs questionnaire, and technical protocols to implement the systematic data collection. These tools will be available for other children’s hospitals at the European level within the ECHO network and beyond, who can easily join the children PREMs observatory in the future.

## Methods

### Overview

The project will use an integrated mix of qualitative and quantitative methods that “seeks convergence, corroboration, correspondence of results from the different methods” [35, p. 259]. It provides a more complete picture, seen from different perspectives, so supporting a more in-depth understanding of the phenomenon. Within a mixed methods strategy, the adoption of different methods of data collection will be used as a means of advancing the analysis, with the results of one method at an earlier stage of research being used to inform the following one, introduced sequentially [35]. Indeed, for each phase of the project, a specific approach, as described below, will be used for producing evidence to be used in the following phases: (1) literature review; (2) analysis of the previous experiences of pediatric PREMs; (3) Delphi process; (4) focus groups (FGs) or in-depth interviews (IDIs) with children and their parents; (5) workshops with interactive working groups; and (6) cross-sectional observational survey.

The project will be based on the participatory action research approach [36,37]. Practitioners and scholars will promote the direct patient participation in research activities [38]. The children’s involvement in all project steps, from the definition of the tool to collect their experience to the definition of information materials and training programs, will allow them to express their views.

The collaboration between university and children’s hospitals will ensure the integration of the scientific approach and interest in new knowledge with the push toward action and engagement to improve the real life of children during their experience of hospitalization. The partnership of the consortium members with key research players, such as the Picker Institute, will support the rigor of the adopted methods. Researchers, hospital managers, and professionals with clinical competences will work together to understand general and context-specific opportunities and challenges, and to support the organizations to make changes in their services to improve the children’s experience. In addition, the direct target of the project, such as children and adolescents, will be engaged directly thanks to the involvement activities carried out by the children’s hospitals.

The project will be inspired by the human rights–based approach to health care [6], aiming to ensure availability, accessibility, acceptability, and quality of health services, specifically hospitalization services for pediatric patients. The principles the project will refer to are nondiscrimination, accountability for health services, as well as enhancing patient-centered health care addressing inequalities. Accordingly, the project will focus not only on outcomes, but also on processes, in particular, assessment and analysis, priority setting, program planning and design, implementation, and monitoring, facilitated by the support of the PREMs that will be implemented. In this way, the project will strengthen the capacities, on the one hand, of the pediatric patients as rights holders to make their claims and, on the other one, of the children’s hospitals as duty bearers to meet their obligations and ensure health care of good quality, using the domains of patient-centered care as a basis [39] and catering for the specific needs of children.

The 2 aforementioned approaches will pervade the methods used in each project phase to achieve their objectives, as will be described in the following sections.

### Literature Review

A systematic scoping review, based on peer-reviewed articles, gray literature, and project reports, will be conducted with the aim of drawing from the literature evidence on the collection of children’s and adolescents’ reported experience of hospitalization. Inclusion criteria, guiding the definition of the search algorithm, will be (1) as specific setting, the hospital stay; (2) as specific target, children and adolescents; (3) PREMs, thus excluding satisfaction and patient-reported outcome measures; (4) tools to collect data on children’s experience, by identifying mechanisms that are aimed at giving children the right to express their voices; (5) use of children’s feedback for quality improvement, in particular at a micro level within a health care organization, namely, PREMs data reporting at the ward level. The results of the literature review will inform the following collaborative process, including the Delphi process, as well as the FGs and IDIs.

### Analysis of the Previous PREMs Experiences in Children’s Hospitals

The project encompasses the analysis of the previous experiences of children PREMs implementation and data collection in the children’s hospitals where a children’s involvement initiative has been already implemented. This activity will aim at identifying and sharing lessons learnt by the children’s hospitals, by analyzing their current or previous experiences in terms of pros and cons, strengths, and weaknesses, as well as good practices concerning PREMs implementation. The experiences will be described by each hospital using a common grid of analysis and shared during a workshop for comparing findings. Field notes will be taken by the participants and used together with the analytical documents on the previous PREMs experiences as complementary data to be analyzed. The analysis will be performed starting from an exchange of knowledge and practices among the children’s hospitals, particularly on tools and mechanisms they used/are using for listening to children’s voices, and on how to include children’s voices in the hospital’s processes of decision making (ie, health care quality improvement actions, hospitalization services’ co-design processes). Also in this case, the results of the previous PREMs experiences’ analysis will inform the collaborative process, namely, the Delphi process, and the FGs and IDIs.

### The Delphi Process

A collaborative, consensus-building process involving children and their families as well as experts and professionals will ensure the development of the PREMs questionnaire as a common tool among the children’s hospitals. This collaborative process based on a Delphi methodology will be implemented for achieving a multidisciplinary consensus on target populations, experience domains and subdomains, specific questions, and administration tools for gathering children’s voices during their hospital stay. The members of the panel will express their opinions on techniques, mechanisms, and tool for engaging with children and adolescents in a systematic, continuous, and structured way, bringing the perspective of their domain of expertise.

The panel will include at least fifty participants, with different professional backgrounds and profiles, such as researchers, hospital staff, and stakeholders, from the various countries and the supporting bodies involved.

Panel members will be contacted and enrolled by the research team, the 4 children’s hospitals, and other supporting institutions, also involving experts from organizations not directly involved in the project, to ensure a wide participation.

Upon acceptance, the members of the panel will receive a detailed informative pack, based on the results of the literature review, on the analysis of the previous PREM experiences and attend a preparatory event. After the latter phase, the panel members will be emailed an invitation to participate in a survey consisting of structured questions for evaluating the PREMs questionnaire for pediatric patients. Two or three rounds are expected, to define a questionnaire for collecting the pediatric patients’ experience. The Delphi process will also include topics for defining the most appropriate tool to directly engage children and adolescents. A final consensus conference will be organized to finalize the process and release the questionnaire as the key final output.

The output of the Delphi process will represent the baseline of the following action research phases.

### FGs and IDIs

For directly involving pediatric patients and their caregivers, each children’s hospital will organize and conduct at least one FG or IDI with children and parents’ representatives. Because of the COVID-19 pandemic, some partners could not adopt this in-dept methodology or will conduct only IDIs. IDIs provide rich qualitative information, although interactions among children or parents cannot be monitored directly.

The objectives of FGs and IDIs will be (1) to explore dimensions and subdimensions of experience with hospitalization that are relevant from the point of view of pediatric patients and parents; (2) to discuss the most appropriate way to involve children and adolescents in providing direct feedback on their experience of hospitalization; and (3) to validate the questions that will be used for PREMs data collection.

Detailed guidelines will be defined by the PI, to guide the hospital partners in the enrollment of pediatric patients and their caregivers, conducting FGs or IDIs or both, and in the data collection and analysis phases. The target population will be:

- Pediatric patients (4-17 years); younger children can be involved for testing dimensions and questions, according to the guidelines.
- Their parents or caregivers (≥18 years).

The enrollment technique and procedure will consider the complexity and specificity of each hospital population, practices, and context. Each hospital will enroll convenience samples, determined by the availability of patients and caregivers, as allowed by the COVID-19 pandemic.

IDI transcripts, field notes, and documents will be kept in text files.

### Workshops With Interactive Group Activities

The project’s most important tool for meeting the objective of the PREMs observatory implementation will be based on the organization of well-prepared workshops. The workshops will focus progressively on the operative design and implementation of PREMs data collection and sharing, the promotion of the awareness among pediatric patients and their caregivers/guardians as well as among professionals about the relevance of PREMs data collection, the mechanisms for benchmarking the PREMs data and their visualization, and, finally, the strategies for using PREMs results for the children’s hospital quality improvement.

Professionals in different areas (ie, technical, communication, data protection, and management) from each partner of the project will work together under the supervision of the Steering Committee and of the Board responsible for the action to produce practical protocols or guidelines that will guide the activities of the projects. Each partner will identify professionals with expertise in the area that is the object of the action to work together: at least one expert for each partner will be selected because of their professional proficiency on the topic.

The Technical and Data Protection Committee of the project will hold a series of videoconferences and workshops. For each topic, a draft protocol will be prepared and discussed during the workshops, with the aim of incorporating the amendments into the documents and distributing them among partners and projects’ boards. This process will allow the committees to define common protocols to design a web system to collect and report survey data. Moreover, collaborative webinars and workshops will be used to define models and guidelines of information materials and training programs for improvement actions based on the PREMs results. Field notes will be taken during the workshops.

### The PREMs Observatory

The outcome of all the previous research activities will be the implementation of a system for digitally and continuously collecting, reporting, and benchmarking data on pediatric experience with hospitalization. Moreover, the project will contribute to identifying and experimenting an innovative tool for the direct involvement of children and adolescents in the PREMs data collection.

It is expected to collect data on children and their caregivers’ experience during a hospital stay in a systematic way and involve all patients and caregivers like a census [28,29,34,40]. Each hospital will systematically engage pediatric patients (0-17 years old) to request their participation in the PREMs data collection.

During the implementation phase of the survey, for administering and collecting the PREMs questionnaire, the hospitals will be free to adopt the organizational and operational processes they prefer to enroll and engage children in participating in the survey. In any case, each hospital will adhere to all the requirements established and agreed to in the previous phases of the project, to perform the PREMs collection on a continuous basis, that is, the participating hospitals will use a minimum standard set of common questions defined throughout the Delphi process and FGs or IDIs, and the same output in terms of data collection, registration, and management. The shared questionnaire might be integrated with other questions relevant for each children’s hospital context. Data will feed a common data reporting web platform for benchmarking children patient–reported experience indicators among hospitals.

It is expected to accompany the survey implementation process with information and communication activities, to make both children and adults aware of the possibility to report about their experience in the hospital. Health care professionals will be also engaged in the training processes. To this end, the project partners will collaboratively design and produce information and communication materials to inform children and caregivers of the PREMs existence. All materials will be designed to be child friendly. While the design will be collaborative, the executive elaboration will be done directly by each hospital, by translating materials and adapting them to the specific context, also deciding what kind of materials to produce.

The PREMs observatory will also allow the design and test of an innovative tool addressing children and adolescents, appropriate for different age groups and enabling them to participate actively in providing their evaluation of the hospitalization experience.

### Ethical Considerations

As the project will directly or indirectly encompass the interaction with humans, specifically with minors, the ethical aspect will be carefully considered. As mentioned in the “Ethical Approval” section, the general protocol of the VoiCEs project has been submitted to an ethics committee by the PI. In addition, all participating hospitals, which will have direct contact with pediatric patients and caregivers and manage individual-level data, will submit the protocol to their Ethics Committee for the research activities directly involving them. This activity will imply ethics review, exemptions, and approvals, with the definition by each hospital of the informed consent process and documents, and of data confidentiality, ownership, use and purposes, treatments, security measures, and appointments for external privacy officers. One of the outputs of the research is a unique data protection and sharing protocol focused on ethics and privacy issues, based on the EU and national laws, including privacy laws, General Data Protection Regulation (GDPR), and the Convention of the Rights of the Child. This document will be used by each hospital for internally defining the detailed and context-specific ethical aspects of the VoiCEs project.

### Ethical Approval

The protocol of the VoiCEs project has been submitted to the approval of the Ethics Committee of the Sant’Anna School of Advanced Studies and Scuola Normale Superiore of Pisa. The approval was obtained on March 25, 2022 (protocol number 07/2022). All participating hospitals submitted the protocol to their Ethics Committee for the research activities involving them directly.

### Data Management and Oversight

Data collection, processing, and sharing processes will be different for each research activity.

The literature review will encompass the search of evidence using literature databases and sources. Data obtained from the search and the consequent analysis will use digital documents and tables with cells organized into rows and columns, such as Excel (Microsoft Inc), Stata (StataCorp), or SAS files (SAS Institute).

For FGs and IDIs, data will be collected through observations, notes, recordings, transcripts, and reports that the hospitals responsible for the activity will prepare. Data will be collected and elaborated by the PI in a final report that will be provided to Delphi panelists, shared with other partners, and submitted to a qualitative comparative analysis.

The Delphi process will encompass an online form to collect the point of view of the panelists. The panelists will give their point of view by accessing a platform using personal identification credentials that will be provided to them via email. Data from each round will be assembled and sent back anonymously to all panel members. Additional rounds will be organized, as the first one, until consensus is reached.

During the workshops, data will be collected through notes, reports, and minutes. Data will be elaborated in a final document (either a protocol or a set of guidelines).

The PREMs survey will gather data with an online questionnaire, or with specific innovative tools or both, according to the outputs of the previous research activities of VoiCEs. The details concerning the content and the process of administration as well as those on data processing and management procedures will be established using the technical and data-sharing protocols that will be developed collectively by the partner under the supervision of the Technical Board and the Data Protection Board:

- A data-sharing protocol focused on privacy issues, setting out the basis on which patient experience data will be collected and used for subsequent actions. The partners will consider the EU and national laws, including privacy laws, GDPR, and the Convention of the Rights of the Child, to ensure the experience data of children are reported in a legal and ethical manner. This protocol will ensure ownership and buy-in of data collection and sharing processes, and will describe all potential and permitted uses of collected patient data for subsequent actions (eg, performance measurement and evaluation, research activity).
- A technical protocol allowing for the creation of a solid web platform to share data. The technical creation of the web platform will be entrusted to a company under the supervision of the Technical Board. It will be a tool to visualize the data.

The main features of the data reporting platform will be the presentation and visualization of quantitative and, if possible, qualitative data collected using tables and graphics, besides assuring the benchmarking activity between hospitals (or single wards) using specific indicators based on the data collected from each hospital.

After sufficient data have been collected, they will be made available to each hospital, and to each ward of the hospitals, whenever data are enough to guarantee the anonymity of respondents. The web platform functionalities will be shared and approved during a specific web workshop.

The data will be conserved for the duration of the project and for the time necessary to complete the reports and the scientific publications envisaged in the project.

Data from the children PREMs observatory will be conserved permanently, and will be stored on servers located in Europe.

The collection and storage systems (eg, web, platforms, servers) will follow the information and communication technology policies of the PI, and additional details and conditions will be established on the data protection protocol that will be collectively elaborated under the supervision of the Data Protection Board.

## Results

### Literature Review

The main objective of the literature review is to map existing evidence on measuring children’s and adolescents’ experience of hospitalization. The research group has analyzed and organized the results by identifying themes and patterns emerging from existing research. The analysis of peer-reviewed papers, reports, and gray literature has explored the following aspects: type of dimensions and subdimensions of children patients’ experience of hospitalization; list of sources (question banks) and previously used questionnaires; type of methodology of the paper; administration and data collection methods; type of population targets to be engaged, such as children of different age groups or with specific health conditions and needs; countries and languages of the study; and use of data for quality improvement actions.

### Analysis of the Previous PREMs Experiences of Children’s Hospitals

The analysis of the previous experiences in PREMs data collection of the children’s hospitals involved in the project will allow to identify the key aspects of these experiences (ie, start date, duration, method of implementation) and the list of questions already used. A comparison of the dimensions and subdimensions covered by these questionnaires will be made, to identify the diverse questions used to investigate the same area. A SWOT analysis will be finally conducted to synthetize the strengths, weaknesses, opportunities, and threats of the process implementation of the PREMs data collection within the children’s hospitals. Results will be checked with data from other sources of information on the topic (ie, websites, reports, articles) to corroborate the findings [35]. Computer software, possibly SAS or Stata, will be used for qualitative data analysis that will follow the approaches suggested by Bailey [41] and Gioia [42].

### Delphi Process

The feedback and evaluations provided by panelists in each round of the Delphi process through closed- and open-ended questions about the content and form will be analyzed by the research team in a descriptive statistical process with measures of central tendency and variability, and qualitatively to synthesize the key emerging themes. The report of each round will be sent back anonymously to all panel members to share answers and discussions, thereby enabling the participants to reflect on different views and modify their own, until the consensus is reached.

Variables of analysis are expected to be related to the relevance of the following areas, disaggregated for characteristics of panelists:

- target groups’ populations in terms of type, age, disease, access to care;
- phases of the hospitalization journey to be investigated;
- type of data collection tool to be administered;
- time of data collection tool administration;
- dimensions and subdimensions of children patients’ experience of hospitalization;
- preferences in terms of the most appropriate way (ie, an eventual innovative tool) to directly engage children and adolescents in collecting their voices.

The results of the Delphi process will be analyzed by performing descriptive statistics, using software such as Stata. If possible, statistical tests will be performed to provide an estimate of the probability that any association among variables is genuine. For instance, additional *t* tests or *χ*^2^ tests could be performed for investigating any differences in the preferences and opinions of different groups of panelists (ie, different stakeholders, different ages, different countries).

The output of the Delphi process, and of the FGs and IDIs, will inform the following phase with the interactive working group realized during online and in-person workshops.

### FGs and IDIs

The results of the FGs and IDIs will be analyzed and presented by focusing on the following contents: type and relevance of dimensions and subdimensions of pediatric patients’ experience of hospitalization; type of data collection tools more appropriate to engage an active participation of children and adolescents (ie, an innovative tool); and validity of questions in cognitive, psychometric, cultural, and linguistic terms. The primary data will be collected directly by the children’s hospitals realizing the FGs or IDIs. Qualitative data will be analyzed using computer software packages by a group of at least two individuals for each hospital. Before the analysis, the research group will define the relevant categories of analysis and the key issues and concerns, also in the form of keywords associated with the themes [35]. In a first phase, the analysis of the qualitative data will be aimed at identifying key aspects for each aforementioned category by target population. Extracts from IDIs and FGs will also be reported in each hospital reports. Each hospital will be responsible for this first analysis of the results and for sharing them with the other partners in a brief report in English. In a second phase, researchers will look for relationships and connections in data from different hospitals, trying also to report the results in diagrams showing the link between concepts and adding some transparency to the process of qualitative data analysis [35]. Finally, the results of the FGs or IDIs will be merged with those of the Delphi process, to ensure the centrality of the children’s and adolescents’ perspectives in the consensus building process on the identification of PREMs data collection tools. This second phase will involve the PI who will conduct a comparative analysis among the results of the 4 hospitals to value the contribution of children’s and caregivers’ involvement.

### Workshops With the Interactive Working Groups

The process of action research implemented during the workshops is based on discussion, comparison, evaluation, and critical analysis of practices by experts to produce an improvement in the field of experience. Before each workshop, the researchers will define the most important contents to be registered and eventually coded (ie, the type of event, participants, shades of opinion, use of a particular word or expression, references provided by participants) [35]. The analysis of these qualitative data could also be conducted using software such as NVivo (QSR International) for facilitating both organization, storage, coding, and analysis of data. The results of this research work will allow the definition of shared documents containing an analysis and interpretation of the contents shared during the workshop, which will act as a base for shared protocols and structured rules of the project.

In addition, quantitative variables will be used to measure the results of the interactive group activities, which will allow to compute process indicators related to (1) the number of people involved and variability of their profile, (2) time spent versus time expected for producing the outputs, and (3) the number of protocols and guidelines finalized.

### The PREMs Observatory

The PREMs observatory will include a questionnaire measuring the experience. This latter questionnaire will be designed according to what emerged during the collaborative process. Each children’s hospital is responsible for the data collection and will send aggregated data to the PI, who will realize comparative analyses and benchmarking on secondary data.

The variables used will measure both process and outcomes [43]. The results of the activity in terms of process will be measured by considering the hospitalization rates and the expected response percentage in each of the children’s hospitals involved in the project. The outcome will be measured in terms of PREMs, distribution among hospitals, and their trend over time in accordance with the improvement actions that will be implemented in each hospital.

The innovative tool will be considered as an additional result, and the feedback from children and adolescents during its experimental use will be analyzed.

## Discussion

### Expected Findings

The VoiCEs project will provide room for innovation, by contributing to the creation of an international and systematic, pediatric PREMs observatory that can be joined by other children’s hospitals or hospitals with pediatric patients, foreseeing the return of usable and actionable data in benchmarking.

Among the expected results, the project will allow:

- the implementation of a European observatory on pediatric PREMs;
- the collection and benchmarking of the pediatric patients’ voices;
- the design and test of innovative methodologies and tools for capturing the pediatric patients’ feedback directly, avoiding caregivers’ intermediation; and
- for a more children- and adolescent-driven health care services’ design.

The VoiCEs project will contribute to enhancing direct participation of children and adolescents in the evaluation and improvement of health care services during hospitalization, considering the scarcity of systematic processes of coassessment [12,13,44]. The project will directly involve young individuals, in addition to adults with various expertise, knowledge, and skills, in a circular process of coassessment for co-design and coinnovation. Indeed, coproduction processes have the most potential in terms of meaningful, effective, and sustainable participation of young people [8]. The final aim is to put in place a system for improving service quality, improving the experience of young people with services, and enhancing the adherence to the principles of public governance with young populations, for which feasibility is not easily or always possible [45].

This project is contributed by the study of the most effective methodologies for collecting the perspectives of children and adolescents, with caution exercised for the customization of tools, techniques, and processes considering their abilities and preferences. Studies have shown that the ability to participate and the opportunity for participation are key antecedents that organizations should consider when designing these processes of direct engagement [15-18]. Moreover, a recent review of the literature showed that the lack of time availability seems to constrain coproduction [44]. Thus, directly involving children and adolescents, who usually have lower limitations in time availability, could be an opportunity to increase the participation of individuals in assessing and designing services better.

The first evidence collected on this topic shows that previous experiences are experimental or single shot [22-25], or struggled in really involving children and adolescents, mainly engaging with parents, particularly mothers [28]. This project will reach its goals by also developing a common standard to be adopted by pediatric hospitals, or hospitals with pediatric patients, for collecting comparable data and activating learning processes among providers.

This use of the results of pediatric patients’ and caregivers’ coassessment can activate a positive circle, by comparatively emphasizing problems or areas for improvement and good practices to learn [10, p. 772]. This implies a prospective use of these data for the future co-design, coinnovation, or codelivery of services [11,12,46]. Benchmarking of these data provides suitable feedback for professionals to enable improvement and reputational mechanisms [47,48], as well as identifying the good performers and the good practices to be disseminated [49].

Another key additional step will be sharing the impact that these data had on the practice. While there is conflicting evidence on the effect of public disclosure on citizen behavior [50]; thus, future research should study people's preferences with and reaction to the exposure to different forms of information based on patient data.

From this perspective, providing organizations with evidence on how to really ensure participation of young people in coassessment of services is extremely relevant for allowing a concrete engagement of the most fragile segments of the population who might be disadvantaged in expressing their opinions, experiences, or preferences. In this sense, this project will contribute to strengthening “the right of the child to be heard” [1].

### Limitations

The project encompasses a coordinated and integrated use of different research methodologies. Their coordination over time is a critical aspect. The research group and the partners from each hospital will identify and minimize issues related to each research approach and methodology.

For the FGs, IDIs, and Delphi process, a principal bias that can be anticipated using relatively small explorative samples is the lack of compliance with the selection criteria, due to the availability of different profiles and the critical circumstances resulting from the pandemic. Concerning the FGs and IDIs, the heterogeneity of feedback, preferences, and needs can be achieved by ensuring the presence of subgroups among the participants, based on relevant characteristics such as age, sex, citizenship, socioeconomic level, and health problems.

In the case of Delphi panelists, special attention will be focused on the representation of different countries, at least on those from which the participating institutions originated (ie, Italy, Latvia, Finland, and the Netherlands); further, owing to the participation of the ECHO network, we will be able to focus on other European countries. Moreover, we will consider different professions and profiles to ensure heterogeneity of opinions and a variety of perspectives. An oversampling of panelists will be performed for minimizing the eventuality of a low response rate.

The PREMs observatory implementation will have to face the obstacles and constraints resulting from the specific contexts and practices already in use, as well as from different policies of data protection and sharing.

### Conclusions

Coassessment may contribute to cocreating public value at different levels [51]. However, it is essential to put in place adequate opportunities to assure people the rights of participating in evaluation initiatives. This is particularly important for vulnerable people in delicate moments. This protocol presents the VoiCEs project, its methodology, and expected results. It aims at studying, identifying, and sharing cocreated methodologies and tools for facilitating the participation of young people in the evaluation and improvement of health care services.

The VoiCEs project will lead to the development of a European observatory on children’s and adolescents’ voices, especially regarding their experience of hospitalization, which would represent a fundamental step in facilitating pediatric patients’ and their caregivers’ participation in coassessment and, lastly, coinnovation processes in health care.

## Abbreviations

AOPI: Italian Association of Children’s Hospitals
AOU Meyer: Meyer Children’s University Hospital
CCUH: Children’s Clinical University Hospital
ECHO: European Children’s Hospitals Organisation
EMC: Erasmus University Medical Center
ERYICA: European Youth and Information Counselling Agency
EU: European Union
FG: focus group
GDPR: General Data Protection Regulation
HCAHPS: Hospital Consumer Assessment of Healthcare Providers and Systems Survey
HUS: HUS New Children’s Hospital
IDI: in-depth interview
PI: principal investigator
PREM: patient-reported experience measure
UNICEF: United Nations International Children’s Emergency Fund/United Nations Children’s Fund
VoiCEs: Value of including the Children Experience for improving their rightS during hospitalization
WHO: World Health Organization

## Data Availability

Data generated or analyzed during this study will be included in future published articles. Moreover, data collected and compared among providers will be published on a public section of the project website [52] as an output of the project itself.
